# Muscle and Adipose Wasting despite Disease Control: Unaddressed Side Effects of Palliative Chemotherapy for Pancreatic Cancer

**DOI:** 10.3390/cancers15174368

**Published:** 2023-09-01

**Authors:** Pamela N. Klassen, Vickie Baracos, Sunita Ghosh, Lisa Martin, Michael B. Sawyer, Vera C. Mazurak

**Affiliations:** 1 Department of Agricultural, Food & Nutritional Sciences, University of Alberta, Edmonton, AB T6G 2R3, Canada; 2 Department of Oncology, University of Alberta, Edmonton, AB T6G 2P5, Canada; 3 Nutrition Services, Alberta Health Services, Edmonton, AB T5J 3E4, Canada

**Keywords:** pancreatic ductal adenocarcinoma, cancer cachexia, skeletal muscle, adipose, chemotherapy, wasting, computed tomography

## Abstract

**Simple Summary:**

Muscle and fat losses during chemotherapy for advanced pancreatic cancer are highly prevalent and associated with tumour progression during treatment. Whether chemotherapy treatment also drives these losses is unknown. This retrospective study aimed to define the effects of chemotherapy and tumour progression on muscle and fat loss and determine the relevance of these losses to overall survival. In a cohort of 210 patients over a standardised scan interval, we demonstrated that tumour progression was associated with greater loss of each tissue compared to tumour control. Furthermore, fluorouracil-based triplet chemotherapy was associated with more muscle loss, while gemcitabine-based doublet chemotherapy was associated with more fat loss. Independent of tumour response, patients with the most muscle or fat loss by tertiles had 72–73% greater hazard of death compared to those with the smallest losses. Muscle and adipose losses are adverse effects of chemotherapy treatment that require targeted management strategies.

**Abstract:**

Muscle and adipose wasting during chemotherapy for advanced pancreatic cancer (aPC) are associated with poor outcomes. We aimed to quantify the contributions of chemotherapy regimen and tumour progression to muscle and adipose wasting and evaluate the prognostic value of each tissue loss. Of all patients treated for aPC from 2013–2019 in Alberta, Canada (*n* = 504), computed-tomography (CT)-defined muscle and adipose tissue index changes (∆SMI, ∆ATI, cm^2^/m^2^) were measured for patients with CT images available both prior to and 12 ± 4 weeks after chemotherapy initiation (*n* = 210). Contributions of regimen and tumour response to tissue change were assessed with multivariable linear regression. Survival impacts were assessed with multivariable Cox’s proportional hazards models. Tissue changes varied widely (∆SMI: −17.8 to +7.3 cm^2^/m^2^, ∆ATI: −106.1 to +37.7 cm^2^/m^2^) over 116 (27) days. Tumour progression contributed to both muscle and adipose loss (−3.2 cm^2^/m^2^, *p* < 0.001; −12.4 cm^2^/m^2^, *p* = 0.001). FOLFIRINOX was associated with greater muscle loss (−1.6 cm^2^/m^2^, *p* = 0.013) and GEM/NAB with greater adipose loss (−11.2 cm^2^/m^2^, *p* = 0.002). The greatest muscle and adipose losses were independently associated with reduced survival (muscle: HR 1.72, *p* = 0.007; adipose: HR 1.73, *p* = 0.012; tertile 1 versus tertile 3). Muscle and adipose losses are adverse effects of chemotherapy and may require regimen-specific management strategies.

## 1. Introduction

Pancreatic cancer (PC) is a leading cause of cancer-related death worldwide, most often diagnosed at an unresectable stage and treated with palliative-intent chemotherapy [1,2,3]. Advanced PC (aPC) often induces severe weight loss that accelerates throughout the disease trajectory [4,5]. Weight loss represents a combination of muscle and adipose loss, which can be accurately measured using sequential computed tomography (CT) images routinely acquired during cancer treatment [6,7,8]. Recent reports describe severe muscle loss in the first 80 days of palliative-intent chemotherapy for PC equivalent to that experienced during 120 days of strict bedrest or a 10-day ventilated critical care hospitalisation [9,10,11,12]. Contrary to the intended effects of palliative chemotherapy, these losses are distressing for patients and may impact both quality of life and survival [9,10,13,14,15,16]. Despite alarming rates of loss described in the literature, drivers of this wasting remain unclear. Some propose that muscle wasting is mainly associated with tumour progression [17,18], leading to assumptions that effective tumour-directed therapy can prevent muscle loss. Others suggest that chemotherapy itself can induce wasting [6,9,19,20]. Babic et al. reported that patients with advanced pancreatic cancer on fluorouracil-based chemotherapy lost more muscle compared to those on gemcitabine or gemcitabine combination therapy (−7.6 cm^2^ vs. −3.6 cm^2^ per 30 days) [9]. Similarly, Carnie et al. demonstrated that patients on FOLFIRINOX were more likely to develop weight loss ≥5% after 4 weeks of treatment compared to patients on gemcitabine or gemcitabine combination therapy [19]. Sex- and BMI-specific risk factors have also been proposed [9,21,22,23]. These hypotheses have been based largely on univariable analyses, leaving the relative contributions of tumour progression and chemotherapeutic agents to muscle and adipose wasting unclear.

Identifying drivers of wasting is highly relevant considering reported associations between muscle loss and reduced survival in advanced PC [9,10,15,24,25]. However, most survival analyses to date have not accounted for key covariates, including concurrent disease progression and loss of adipose tissue. Methodological variability also exists in the period of time over which tissue change is measured and in the treatment of muscle or adipose wasting as variables in survival analysis. In short, no single multivariable analysis has deconvoluted survival impacts of commonly observed muscle and total adipose losses while accounting for concurrent disease response over a clearly defined period of treatment for aPC. 

The primary aim of this study was to define the independent impacts of chemotherapy treatment and tumour progression on magnitude of muscle and adipose loss in the first 3 months of chemotherapy for aPC, using a linear regression-based approach. The secondary aim was to confirm the prognostic relevance of these muscle and adipose losses, adjusted for tumour response. The two most common palliative-intent regimens for aPC are FOLFIRINOX (5-fluorouracil, folinic acid, irinotecan, and oxaliplatin) and gemcitabine plus *nab*-paclitaxel (GEM/NAB), prescribed at the discretion of the medical oncologist based on factors including age, performance status, and patient preference [2,3]. We hypothesised that FOLFIRINOX chemotherapy compared to GEM/NAB and disease progression compared to disease control would be independently associated with greater-magnitude muscle and adipose losses.

## 2. Materials and Methods

**Figure 1:**
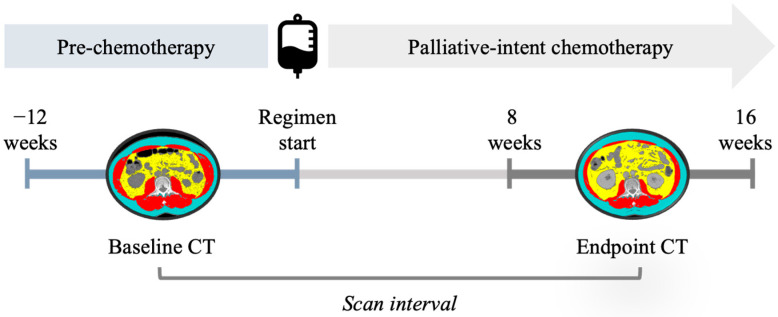
Criteria for inclusion of routinely acquired computed-tomography (CT) scans for quantification of change in skeletal muscle and adipose tissue during initial 12 ± 4 weeks of palliative chemotherapy. Scan interval: time between selected CT scans.

All adult patients (≥18 years of age) who underwent first-line palliative-intent chemotherapy with either FOLFIRINOX or GEM/NAB for unresectable locally advanced or metastatic pancreatic ductal adenocarcinoma in Alberta between 1 January 2013 and 31 December 2019 were identified retrospectively by data request from the Alberta Cancer Registry [2,3]. Patients were included in the present analysis if they had analysable CT scans at the third lumbar vertebra (L3) at both baseline (before palliative chemotherapy) and endpoint (disease re-assessment). Baseline was defined as the CT scan closest to the palliative regimen start date (up to 12 weeks prior), and endpoint as the CT scan closest to 12 weeks from regimen start (±4 weeks) (Figure 1). Pre-defined scan timing was intended to standardise the time and exposure to chemotherapy during which change was measured. Further, scan timing was selected to include the maximum number of patients in whom initial palliative-intent treatment response could be ascertained and increase the likelihood of observing detectable change above measurement error.

The axial CT slices at the centre of the third lumbar vertebra (L3) were selected on baseline and endpoint scans using a split screen to ensure consistent anatomical location. Body composition analysis was undertaken using CT scans according to methods previously described [7]. In short, axial images were auto-segmented using the ABACS module of Slice-O-Matic (Tomovision, Montreal, Canada) according to predefined Hounsfield unit (HU) thresholds to delineate skeletal muscle (SM, −29 to +150 HU) and adipose tissue (AT, −30 to −190 HU). Subcutaneous, visceral, and intermuscular adipose tissue areas were summed to determine total adipose tissue area. Margins were manually corrected by two trained observers according to a defined protocol. A single observer corrected both scans for any individual patient, limiting inter-observer variability in longitudinal analysis of change. 

**Figure 2:**
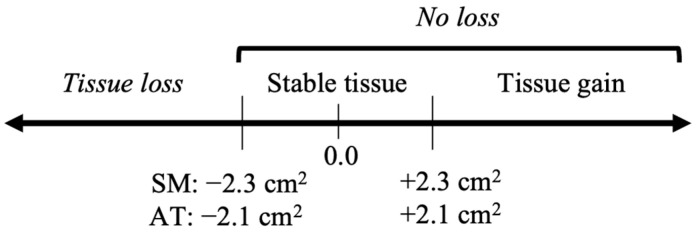
Definition of least significant change (LSC, cm^2^) in skeletal muscle (SM) and adipose tissue (AT) area at the L3 vertebra on axial computed-tomography images based on measured precision error of the observers.

A precision test was completed by each observer prior to analysis, consisting of 30 unidentifiable images manually analysed twice by the same observer at least 24 h apart to calculate the least significant change (LSC) value for each observer [26]. The largest LSC among two observers was 2.3 cm^2^ for skeletal muscle and 2.1 cm^2^ for adipose tissue. Patients who lost more muscle than the LSC value (2.3 cm^2^) were classified as having *muscle loss*; similarly, those who lost more adipose than the LSC value (2.1 cm^2^) were classified as having *adipose loss* (Figure 2). 

Cross-sectional areas of muscle and adipose (cm^2^) at L3 were normalised for height and reported as skeletal muscle index (SMI) and adipose tissue index (ATI) in cm^2^/m^2^ [27]. Absolute change for each tissue was calculated as endpoint minus baseline value. Relative change (%) was calculated by dividing absolute change by baseline value and multiplying by 100. 

Demographic and clinical data including age at baseline, biological sex, first palliative-intent chemotherapy regimen, disease stage at palliative regimen start (locally advanced or metastatic/recurrent), topography (tumour location in pancreas: head/neck or body/tail), prior surgical resection, primary treatment centre, treatment dates, height, weight at the time of each CT scan, and date of death were collected by data request from the Alberta Cancer Registry and electronic medical records. Patients included with recurrence all had undergone prior surgical resection with adjuvant chemotherapy before presentation for palliative chemotherapy. Tumour response between baseline CT and endpoint CT was acquired from the electronic radiologist report: stable disease or partial/complete response were considered *tumour control*, whereas progressive disease or mixed response (i.e., discordant response between primary tumour and metastases) were considered *tumour progression*. Treatment after the endpoint CT was classified as no further treatment, ongoing palliative chemotherapy, or curative resection. Treatment after the endpoint CT was determined at the discretion of the oncologist in partnership with the patient. Generally, patients with disease progression who were not fit for the alternate regimen had no further treatment (i.e., received best supportive care); fit patients with disease progression switched to the alternate regimen. Patients with disease control continued their first line of therapy, except in unusual circumstances. In rare cases, patients with sufficient disease response to initial therapy underwent curative resection followed by adjuvant chemotherapy. Dates of death were confirmed in the electronic medical record, and patients found to be alive at the time of the search were censored using the date of the most recent oncological visit or CT scan. Overall survival (OS) was calculated in terms of days from palliative-regimen start date to death.

Baseline characteristics and changes in muscle, adipose, and weight were compared between regimens using Pearson’s chi-squared tests for categorical variables and independent t-tests for continuous variables. For regression modelling, SMI and ATI changes were normalised to the median scan interval (115 days) to account for potential impact of time. Linear regression was used to identify factors associated with muscle or adipose change, in which SMI change and ATI change were treated separately as dependent variables. At the univariable level, age, sex, metastatic disease at baseline, presence of the primary tumour, tumour topography, treatment regimen, tumour response at endpoint CT, and baseline body mass index (BMI, per 5 kg/m^2^) were examined; those values significant at *p* < 0.20 were entered into multivariable analysis.

The impacts of muscle and adipose changes on OS were explored in two multivariable Cox’s proportional hazard models. In the first, continuous muscle and adipose changes were used as predictors to demonstrate the incremental survival impact of small changes. Continuous muscle and adipose changes were scaled so that 1 unit of change was equal to −2.0 cm^2^/m^2^ for muscle and −10.0 cm^2^/m^2^ for adipose; this scaling was performed to ensure that the continuous units in the survival model were larger than the margin of error and approximately proportionate to each other in terms of mean change magnitudes. A second model was developed using tertiles of change to demonstrate the risk associated with the greatest losses, using top tertile change (i.e., T3, gain or mild loss) as the reference. Additional variables tested included age at baseline, sex, metastatic disease at baseline, treatment regimen, tumour response at endpoint CT, and subsequent treatment after endpoint CT. Factors significant at *p* < 0.20 on univariable analysis were entered into the multivariable models [28]. Interactions between variables were included stepwise in the models, with none found to be significant. Statistical analyses were completed with IBM SPSS Statistics 25.

## 3. Results

### 3.1. Participants

Of 504 patients who received standard palliative-intent chemotherapy for PC in Alberta from 2013–2019, 210 met inclusion criteria for this analysis (Figure S1). Cohort characteristics and clinical outcomes are presented in Table 1. Males represented 54% of the cohort, with a median age of 64 years; tumours were primarily metastatic or recurrent (67.1%) and located in the pancreatic head/neck (59.5%). GEM/NAB was the most common regimen (57.1%), and patients were equally distributed between the two tertiary treatment centres. Patients on GEM/NAB were significantly older than those on FOLFIRINOX (*p* < 0.0005); disease characteristics and baseline body composition were not significantly different between regimens. Although the entire cohort was treated initially with palliative intent, 11 (5.2%) had sufficient disease response to enable resection after the endpoint CT scan. A significantly higher proportion of patients on FOLFIRINOX underwent further palliative chemotherapy or curative resection after the endpoint CT.

### 3.2. Magnitude and Drivers of Muscle and Adipose Change

**Figure 3:**
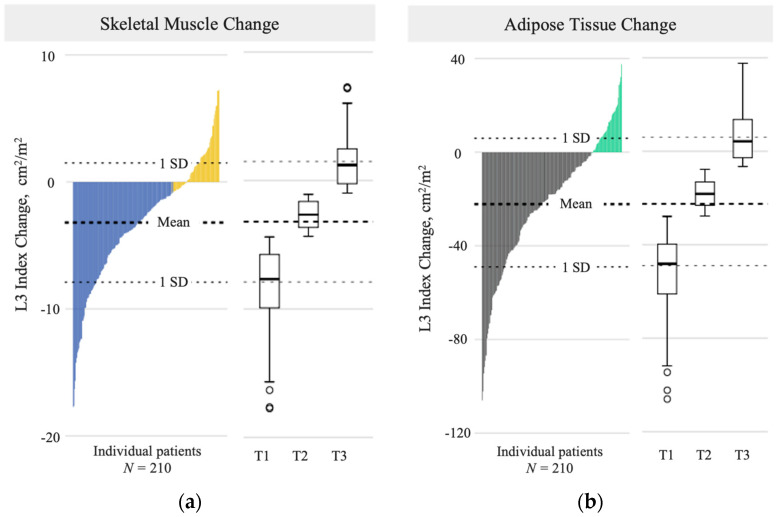
(**a**) Skeletal muscle index change over 116 (23) days, represented by a waterfall plot (left) and tertile boxplots (right); mean overall change: −3.2 (4.7) cm^2^/m^2^; blue: *muscle loss*; yellow: *no muscle loss*; (**b**) adipose tissue index change over 116 (23) days, represented by a waterfall plot (left) and tertile boxplots (right); mean overall change: −21.5 (27.5) cm^2^/m^2^; grey: *adipose loss*; green: *no adipose loss*; L3: third lumbar vertebra; T: tertile.

The mean scan interval between the baseline and endpoint CT was 116 (27) days and did not differ by regimen (Table 2). Muscle change (∆) ranged from −17.8 cm^2^/m^2^ to +7.3 cm^2^/m^2^, with muscle loss (greater than the LSC) occurring in 68% of the cohort (Figure 3a). SMI change tertiles included T1: ∆ ≤ −4.3 cm^2^/m^2^; T2: ∆ −4.2 to 1.1 cm^2^/m^2^; and T3: ∆ ≥ −1.0 cm^2^/m^2^. Males on FOLFIRINOX had greater mean muscle loss than males on GEM/NAB (*p* < 0.05); there was no regimen-based difference in mean muscle change among females (Table 2). Adipose change ranged from −106.1 cm^2^/m^2^ to +37.7 cm^2^/m^2^, with adipose loss (greater than the LSC) observed in 77% of the cohort (Figure 3b). ATI change tertiles included T1 (severe loss): ∆ ≤ −27.7 cm^2^/m^2^; T2 (moderate loss): ∆ −27.6 to −7.7 cm^2^/m^2^; and T3 (mild loss, or gain): ∆ ≥ −7.6 cm^2^/m^2^. Both males and females on GEM/NAB lost more adipose than those on FOLFIRINOX (Table 2; *p* = 0.006, males and *p* = 0.029, females). Concurrent muscle and adipose loss occurred in 57% of patients. Additional metrics describing the change observed are available in Table S1.

In the multivariable linear regression model for muscle change (Table 3), factors significantly associated with greater muscle loss included tumour progression (−3.2 cm^2^/m^2^ vs. *tumour control*), FOLFIRINOX regimen (−1.6 cm^2^/m^2^ vs. *GEM/NAB*), male sex (−1.3 cm^2^/m^2^ vs. *female*), and higher baseline BMI (−1.2 cm^2^/m^2^ per 5 kg/m^2^). Metastatic disease at baseline and head/neck tumour topography were significant factors in the univariable analysis but were not significant in the multivariable model (adjusted R^2^ 0.192, *p* < 0.001). 

The multivariable linear regression model for adipose change (Table 3) revealed that tumour progression and higher baseline BMI were significantly associated with more adipose loss (−12.4 cm^2^/m^2^ vs. *tumour control* and −6.9 cm^2^/m^2^ per 5 kg/m^2^, respectively). FOLFIRINOX treatment was associated with less adipose loss (+11.2 cm^2^/m^2^ vs. *GEM/NAB*). The presence of the primary tumour and head/neck tumour topography were significant factors in the univariable analysis but did not reach significance in the multivariable model (adjusted R^2^ 0.154, *p* < 0.001).

### 3.3. Survival Impact of Muscle and Adipose Losses

At the time of analysis, 95% of patients were deceased. In the multivariable Cox’s proportional hazards model using continuous muscle and adipose changes as predictors (Model 1, Table 4), muscle change per −2 cm^2^/m^2^ and adipose change per −10 cm^2^/m^2^ were independently associated with reduced OS (HR 1.10, [95% CI 1.04, 1.18], *p* = 0.003 and HR 1.10, [95% CI 1.03, 1.17], *p* = 0.003) after adjustment for tumour progression, sex, and subsequent treatment after the endpoint CT. In the multivariable model using tertiles of muscle and adipose change as predictors (Model 2, Table 4), the lowest tertile muscle and adipose changes (i.e., severe losses) were independently associated with reduced OS (HR 1.72, [95% CI 1.16, 2.57], *p* = 0.007 and HR 1.73, [95% CI 1.13, 2.66], *p* = 0.012). Tumour progression (versus control) was associated with increased hazard of death while subsequent palliative chemotherapy or curative resection (versus no further treatment) was associated with reduced hazard of death in both models. The final models contained no significant interactions and were sufficiently powered to avoid overfitting. An additional model was developed, using continuous muscle and adipose changes per estimated 1 kg of total body tissue loss (Table S2) [7], in which the effects and significance of continuous muscle and adipose changes were consistent with those observed in Table 4, regardless of the metrics used to quantify tissue loss.

## 4. Discussion

This study employed a multivariable regression-based approach to clarify unique contributions of chemotherapy regimen and tumour progression to muscle and adipose wasting during early palliative chemotherapy for aPC. We observed wide variability in CT-defined muscle and adipose tissue change over a standardised time interval, ranging from large losses to moderate gains, consistent with past reports [9,10,15]. Tumour response, chemotherapy regimen, sex, and baseline BMI significantly contributed to variability in the magnitude of muscle and adipose change.

Early efforts to measure muscle change in patients with cancer suggested a relationship between tumour progression and muscle loss, but the magnitude of this relationship was unknown [17,18]. In our cohort, disease progression was independently associated with 3.2 cm^2^/m^2^ more muscle loss and 12.4 cm^2^/m^2^ more adipose loss versus disease control over 115 days, independent of other significant factors. Mechanisms behind disease-driven wasting include nutrient consumption by a tumour, tumour-induced catabolic and lipolytic signals, systemic inflammation, and tumour-induced appetite loss [29,30]. Specific to PC, tumour growth may correspond to the progressive destruction of the endocrine and exocrine digestive functions of the pancreas, leading to malabsorption and malnutrition [31,32]. 

Tumour progression must be accounted for as a high-magnitude contributor to muscle and adipose loss in advanced PC, but importantly, it is not the sole driver of wasting. Our analysis demonstrates, for the first time, that two common chemotherapy regimens for aPC have unique independent effects on muscle and adipose tissue. FOLFIRINOX chemotherapy was associated with 1.6 cm^2^/m^2^ more muscle loss compared to GEM/NAB, while GEM/NAB was associated with 11.2 cm^2^/m^2^ more adipose loss. These treatment-specific losses are not inconsequential, as demonstrated by survival analysis. Muscle loss per 2 cm^2^/m^2^ and adipose loss per 10 cm^2^/m^2^ over the first 115 days of chemotherapy were each associated with 10% greater hazard for death, independent of tumour response and subsequent treatment. Considering that muscle loss up to 17.8 cm^2^/m^2^ and adipose loss up to 106.1 cm^2^/m^2^ were observed in this cohort, with 57% of patients losing both tissues concurrently, the impact of moderate losses cannot be ignored. Survival analysis by tertiles of tissue change revealed that the greatest losses of muscle and adipose were independently associated with greater hazard for death (HR 1.72 and HR 1.73, respectively) compared to tissue gain or mild loss, independent of tumour response. The management of these early treatment side effects to promote muscle and adipose maintenance should be of high priority during palliative chemotherapy, following evidence-based recommendations for clinical practice. Specifically, the medical management of nutrition-impact symptoms (e.g., nausea, exocrine insufficiency) alongside interventions to optimise protein and energy intake, inhibit systemic inflammation, and increase physical activity are recommended to attenuate wasting [33]. 

Mechanisms of regimen-specific effects on tissue could be related to treatment side effects (impacting nutritional intake) or direct tissue toxicity. Patients on FOLFIRINOX can experience a more severe side-effect profile compared to GEM/NAB, and differences in the toxicity profile should be investigated as potential explanatory factors for the associations we demonstrate [19,34]. In our cohort, the absence of oral intake data limits a nutrition-oriented hypothesis; however, weight loss was not different between regimen groups. Alternatively, an increasing number of experimental studies provide evidence for the direct effects of anti-cancer agents on skeletal muscle (reviewed by [6,35,36]). Most recently, VanderVeen et al. reported that 5-fluorouracil impaired muscle repair in a mouse model, while Halle et al. found that 5-fluorouracil plus oxaliplatin, two of the main components of FOLFIRINOX, impaired muscle function and reduced muscle mass in mice [37,38]. The direct effects of gemcitabine and/or *nab*-Paclitaxel on adipose tissue have not been explored in the literature, and this is a direction for future research. Mechanistic studies where biologic material is available could be used to evaluate regimen-specific effects on circulating factors affecting lipolysis and proteolysis, leading to tissue-specific wasting, as reviewed by Kadakia et al. [39] and Baracos and Schiessel [36]. 

These results have implications outside of the palliative setting. FOLFIRINOX is used as both a neoadjuvant and adjuvant therapy for resectable pancreatic tumours [40]. Even in the absence of a growing tumour, FOLFIRINOX-related muscle loss during each of these treatment courses may impact recovery, rehabilitation, and survivorship [41,42,43,44], requiring investigation with prospective studies. Further, patients who undergo successful resection for PC often present with recurrent disease and must again withstand chemotherapy. Muscle losses incurred during neoadjuvant and adjuvant treatment may impact tolerance to future palliative-intent chemotherapy [24,45].

In addition to the impacts of disease progression and chemotherapy regimen, we demonstrated that male sex is associated with greater muscle but not adipose loss, while higher baseline BMI is associated with greater loss of both tissues, concurring with prior reports [9,21,23,46]. Contrary to the belief that obese patients ‘have more to lose’, particular attention should be given to monitoring and supporting those with high BMIs, who may have difficulty meeting higher energy requirements in the face of appetite loss or treatment side effects [23]. 

A significant strength of this study was use of repeated CT images to obtain precise measurements of muscle and adipose change during a clearly defined period of palliative chemotherapy. We limited the impact of time in our models by standardising the interval between the baseline and endpoint CT scans and further adjusting observed muscle and adipose change to the median scan interval. The selection of patients with re-evaluation CT scans 3 ± 1 month after the treatment start excluded those with rapid decline in condition or early death, improving the relevance of our results to patients who are evaluated for disease response on a standard schedule. 

With respect to survival analysis, the adjustment for concurrent disease response and subsequent treatment in our model adds strength to the assertion that muscle loss during early palliative therapy impacts OS [9,10,15,16]. Our results conflict, in one sense, with those of Salinas-Miranda et al. and Babic et al., who found no prognostic value associated with adipose tissue loss [9,10]. This disparity could be related to the former studies’ division of adipose tissue into visceral and subcutaneous compartments rather than considering them together; this separation overlooks the potential prognostic power of total adipose tissue loss in relatively small samples of patients. 

The limitations of our study are related to retrospective data availability and the absence of a validation cohort. Linear regression models explained only 20% of the variability in skeletal muscle change and 15% of the variability in adipose tissue change compared to mean models (adjusted R^2^ values: 0.19 and 0.154, respectively). Additional contributors for which data were unavailable include differences in oral intake, malabsorption associated with pancreatic exocrine insufficiency, alterations in endocrine function, tumour-induced metabolic effects, systemic inflammation, cumulative chemotherapy dose and toxicity profile, and genetic variability. These factors should be considered in large prospective studies. Further, with a sample size of 210 patients from a single Canadian province, our results require validation in a larger, more diverse population, with data on cumulative chemotherapy doses received. 

## 5. Conclusions

This study demonstrates that standard palliative chemotherapy regimens contribute uniquely to muscle and adipose wasting in advanced pancreatic cancer independent of disease response. In the first 12 weeks of palliative chemotherapy, FOLFIRINOX led to greater muscle loss while GEM/NAB led to greater adipose loss, representing unaddressed side effects of treatment. Muscle and adipose losses in this period each had an independent survival impact, and greatest losses of each tissue were associated with approximately 75% greater hazard for death compared to mild loss or gain. These impacts would be intensified in patients who lost both tissues, representing 57% of our cohort. Given the implications for clinical outcomes, attenuating muscle and adipose loss during initial chemotherapy should be of high priority in research and practice. Researchers should consider both muscle and total adipose changes as important outcomes and account for treatment regimen and tumour response when evaluating strategies to attenuate wasting. While research continues, our data provide the clinician with accessible criteria to identify patients at risk of wasting, along with evidence to support close monitoring and proactive intervention.

## Figures and Tables

**Table 1:** Cohort characteristics.

	Overall	FOLFIRINOX	GEM/NAB
**Demographics**			
Number of patients, *N (% of cohort)*	210	90 (42.9)	120 (57.1)
Age, years, median (IQR)	64 (58, 70)	61 (57, 66)	68 (59, 73) *
Sex, male	114 (54.3)	40 (55.6)	64 (53.3)
Treatment centre, *N (%)*			
*Centre 1*	106 (50.5)	44 (48.9)	62 (51.7)
*Centre 2*	104 (49.5)	46 (51.1)	58 (48.3)
**Baseline Disease Characteristics**			
Tumour topography, *N (%)*			
*head/neck*	125 (59.5)	48 (53.3)	77 (64.2)
*body/tail*	52 (24.8)	24 (26.7)	28 (23.3)
*overlapping/unspecified*	33 (15.7)	18 (20.0)	15 (12.5)
Disease stage, *N (%)*			
*Locally advanced*, *unresectable*	69 (32.9)	29 (32.2)	40 (33.3)
*Metastatic or recurrent*	141 (67.1)	61 (67.8)	80 (66.7)
Primary tumour, *N (%)*			
*Previously resected*	26 (12.4)	12 (13.3)	14 (11.7)
*Present*	184 (87.6)	78 (86.7)	106 (88.3)
**Baseline Body Composition**			
Skeletal muscle index (cm^2^/m^2^)			
*Male*	49.4 (8.2)	50.5 (8.9)	48.5 (7.6)
*Female*	39.0 (5.9)	39.1 (6.8)	39.0 (5.3)
Adipose tissue index (cm^2^/m^2^)			
*Male*	99.6 (52.8)	91.4 (44.2)	106.1 (58.2)
*Female*	115.5 (65.6)	104.1 (55.0)	123.7 (71.5)
Body mass index (kg/m^2^)			
*Male*	26.2 (4.1)	25.8 (3.6)	26.6 (4.4)
*Female*	25.4 (4.9)	25.1 (4.7)	25.6 (5.2)
BMI WHO classification, *N (%)*			
*underweight*, <*18.5*	6 (2.9)	2 (2.2)	4 (3.3)
*normal weight*, *18.5–24.9*	88 (41.9)	41 (45.6)	47 (39.2)
*overweight*, *25.0–29.9*	80 (38.1)	35 (38.9)	45 (37.5)
*obesity*, ≥*30.0*	36 (17.1)	12 (13.3)	24 (20.0)
**Clinical Outcomes**			
Tumour response at endpoint CT, *N (%)*			
*Tumour control*	137 (65.2)	57 (63.3)	80 (66.7)
*Progression*	73 (34.8)	33 (36.7)	40 (33.3)
Treatment after endpoint CT, *N (%)*			
*No further treatment*	68 (32.4)	19 (21.1) *	49 (40.8)
*Ongoing palliative chemotherapy*	131 (62.4)	63 (70.0) *	68 (56.7)
*Curative resection*	11 (5.2)	8 (8.9) *	3 (2.5)
Overall survival			
Days (median, 95% CI)	377 (335, 418)	409 (342, 476)	349 (304, 394)

* *p* < 0.05, FOLFIRINOX vs. GEM/NAB; values are means (standard deviation) unless otherwise noted. FOLIFIRINOX: multi-agent chemotherapy consisting of 5-fluorouracil, folinic acid, irinotecan, and oxaliplatin; GEM/NAB: doublet chemotherapy consisting of gemcitabine and nab-Paclitaxel; BMI: body mass index; CT: computed tomography.

**Table 2:** Skeletal muscle, adipose tissue, and weight change observed during initial palliative-intent chemotherapy for pancreatic cancer.

	Overall	FOLFIRINOX	GEM/NAB
Baseline CT *(days from regimen start)*	−33 (22)	−33 (22)	−33 (23)
Endpoint CT *(days from regimen start)*	83 (16)	80 (16)	85 (15)
Scan Interval *(days)*	116 (27)	113 (28)	118 (26)
**Skeletal Muscle Index Change (cm^2^/m^2^)**			
*Male*	−3.9 (5.3)	−5.1 * (4.9)	−3.0 (5.4)
*Female*	−2.3 (3.9)	−2.4 (3.6)	−2.2 (4.1)
**Skeletal Muscle Relative Change *(%)***			
*Male*	−7.8 (10.1)	−10.2 * (9.3)	−5.9 (10.5)
*Female*	−5.4 (9.5)	−5.7 (8.8)	−5.2 (10.1)
**Adipose Tissue Index Change (cm^2^/m^2^)**			
*Male*	−20.4 (28.2)	−12.3 (28.3)	−26.8 * (26.6)
*Female*	−22.8 (26.9)	−15.7 (25.9)	−27.8 * (26.7)
**Adipose Tissue Relative Change *(%)***			
*Male*	−17.5 (35.1)	−11.0 (30.0)	−22.7 (38.1)
*Female*	−18.8 (26.6)	−13.6 (25.8)	−22.6 (26.8)
**Weight Relative Change *(%)***			
*Male*	−3.7 (6.6)	−4.8 (6.8)	−2.7 (6.3)
*Female*	−3.7 (6.3)	−2.6 (5.6)	−4.5 (6.7)

* *p* < 0.05, FOLFIRINOX vs. GEM/NAB; values are means (standard deviation) unless otherwise noted. FOLIFIRINOX: multi-agent chemotherapy consisting of 5-fluorouracil, folinic acid, irinotecan, and oxaliplatin; GEM/NAB: doublet chemotherapy consisting of gemcitabine and nab-Paclitaxel; BMI: body mass index; CT: computed tomography.

**Table 3:** Linear regression models identifying factors associated with skeletal muscle and adipose tissue index change (cm^2^/m^2^).

	(a) Association with Skeletal Muscle Index (SMI) Change						(b) Association with Adipose Tissue Index (ATI) Change					
	Univariable			Multivariable			Univariable			Multivariable		
Characteristic	*β*	95% CI	*p*-Value	*β*	95% CI	*p*-Value	*β*	95% CI	*p*-Value	*β*	95% CI	*p*-Value
Male sex *(vs. female)*	−2.02	−3.34, −0.69	0.003	**−1.28**	**−2.53, −0.03**	**0.044**	1.35	−6.02, 8.72	0.719	3.63	−3.26, 10.52	0.300
Metastatic at baseline *(vs. locally advanced)*	−1.28	−2.71, −0.16	0.080	−0.39	−1.74, 0.95	0.562	−0.92	−8.76, 6.92	0.817	-	-	-
Primary tumour present *(vs. previously resected)*	−0.89	−2.94, 1.17	0.396	-	-	-	−10.43	−21.47, 0.61	0.064	−10.14	−20.49, 0.20	0.055
Head/neck topography *(vs. body/tail/unknown)*	0.93	−0.45, 2.31	0.185	0.71	−0.55, 1.98	0.267	-6.24	−13.67, 1.20	0.100	−5.64	−12.62, 1.34	0.112
FOLFIRINOX *(vs. GEM/NAB)*	−1.64	−2.99, −0.29	<0.001	**−1.58**	**−2.82, −0.34**	**0.013**	12.01	5.35, 19.78	0.001	**11.19**	**4.32, 18.06**	**0.002**
Tumour progression *(vs. control)*	−3.57	−4.91, −2.23	<0.001	**−3.22**	**−4.53, −1.92**	**<0.001**	−11.70	−18.85, −3.75	0.001	**−12.39**	**−19.55, −5.23**	**0.001**
Baseline BMI (per 5 kg/m^2^*)*	−1.19	−1.93, −0.45	0.002	**−1.21**	**−1.89, −0.52**	**0.001**	−7.39	−11.35, −3.24	<0.001	**−6.85**	**−10.63, −3.07**	**<0.001**

FOLIFIRINOX: multi-agent chemotherapy consisting of 5-fluorouracil, folinic acid, irinotecan, and oxaliplatin; GEM/NAB: doublet chemotherapy consisting of gemcitabine and *nab*-Paclitaxel; BMI: body mass index. Bold text indicates statistical significance.

**Table 4:** Cox’s proportional hazard models demonstrating association between skeletal muscle and adipose tissue changes and overall survival.

Characteristic	(a) Univariable			(b) Multivariable Model 1			(c) Multivariable Model 2		
	HR	95% CI	*p*-Value	HR	95% CI	*p*-Value	HR	95% CI	*p*-Value
Age *(per year)*	0.99	0.98, 1.01	0.570	n/a	n/a	n/a	n/a	n/a	n/a
Male sex *(vs. female)*	1.10	0.90, 1.59	0.212	0.79	0.61, 1.13	0.132	0.88	0.65, 1.19	0.880
Metastatic *(vs. locally advanced)*	1.50	1.11, 2.02	0.008	1.29	0.94, 1.80	0.119	1.28	0.92, 1.78	0.137
FOLFIRINOX *(vs. GEM/NAB)*	0.70	0.53, 0.93	0.014	1.01	0.742, 1.38	0.942	0.94	0.69, 1.29	0.716
Tumour progression *(vs. tumour control)*	3.04	2.25, 4.13	<0.001	**2.20**	**1.58, 3.04**	**<0.001**	**2.14**	**1.53, 2.99**	**<0.001**
Treatment after endpoint CT:									
no further treatment;	ref			ref			ref		
ongoing palliative chemotherapy;	0.29	0.29, 0.39	<0.001	**0.35**	**0.25, 0.49**	**<0.001**	**0.33**	**0.23, 0.47**	**<0.001**
curative resection	0.06	0.02, 0.14	<0.001	**0.07**	**0.02, 0.20**	**<0.001**	**0.06**	**0.21, 0.19**	**<0.001**
SMI change *(per −2.0 cm^2^/m^2^)*	1.09	1.05, 1.13	<0.001	**1.10**	**1.04, 1.18**	**0.003**	-	-	-
ATI change *(per −10.0 cm^2^/m^2^)*	1.15	1.10, 1.19	<0.001	**1.10**	**1.03, 1.17**	**0.003**	-	-	-
SMI change tertile (cm^2^/m^2^)									
≥−1.0 (T3, gain/maintenance/mild loss)	ref			-	-	-	ref		
−1.1 to −4.2 (T2, moderate loss)	1.26	0.88, 1.77	0.199	-	-	-	1.29	0.90, 1.85	0.168
≤−4.3 (T1, severe loss)	2.20	1.56, 3.11	<0.001	-	-	-	**1.72**	**1.16, 2.57**	**0.007**
ATI change tertile (cm^2^/m^2^)									
≥−7.6 (T3, gain or mild loss)	ref			-	-	-	ref		
−7.7 to −27.6 (T2, moderate loss)	1.55	1.09, 2.21	0.014	-	-	-	1.08	0.73, 1.59	0.696
≤−27.7 (T1, severe loss)	3.01	2.10, 4.30	<0.001	-	-	-	**1.73**	**1.13, 2.66**	**0.012**

ATI: adipose tissue index; CT: computed tomography; SMI: skeletal muscle index; T: tertile; Model 1 Chi-square 140.201, *p* < 0.001; Model 2 Chi-square 138.009, *p* < 0.001. Bold text indicates statistical significance.

## Data Availability

The data presented in this study are available on request from the corresponding author. The data are not publicly available due to the ethical limitations of the waiver of consent.

## Supplementary Materials

The following supporting information can be downloaded at https://www.mdpi.com/article/10.3390/cancers15174368/s1, Figure S1: Flow chart of patient selection; Table S1: Additional metrics describing skeletal muscle, adipose tissue, and weight change from baseline to endpoint; Table S2: Cox’s proportional hazard model demonstrating survival impact of 1 kg estimated total body skeletal muscle mass and adipose tissue mass loss over a median scan interval of 115 days.
