# Supplementary material for: Muscle and Adipose Wasting despite Disease Control: Unaddressed Side Effects of Palliative Chemotherapy for Pancreatic Cancer
Source: Cancers (Basel). 2023 Sep 1;15(17):4368. doi: 10.3390/cancers15174368 (PMC10486774; doi:10.3390/cancers15174368)
Supplement: Supplementary file 1 [file cancers-15-04368-s001.zip › cancers-2529260-supplementary.pdf]

**Figure S1**: Flow chart of patient selection from total cohort (n=504) who initiated palliative-intent chemotherapy in Alberta, Canada from 2013-2019. Patients were excluded if they had no baseline CT image within 90 days prior to treatment start, and/or no follow-up CT within 84 ± 28 days after chemotherapy initiation.

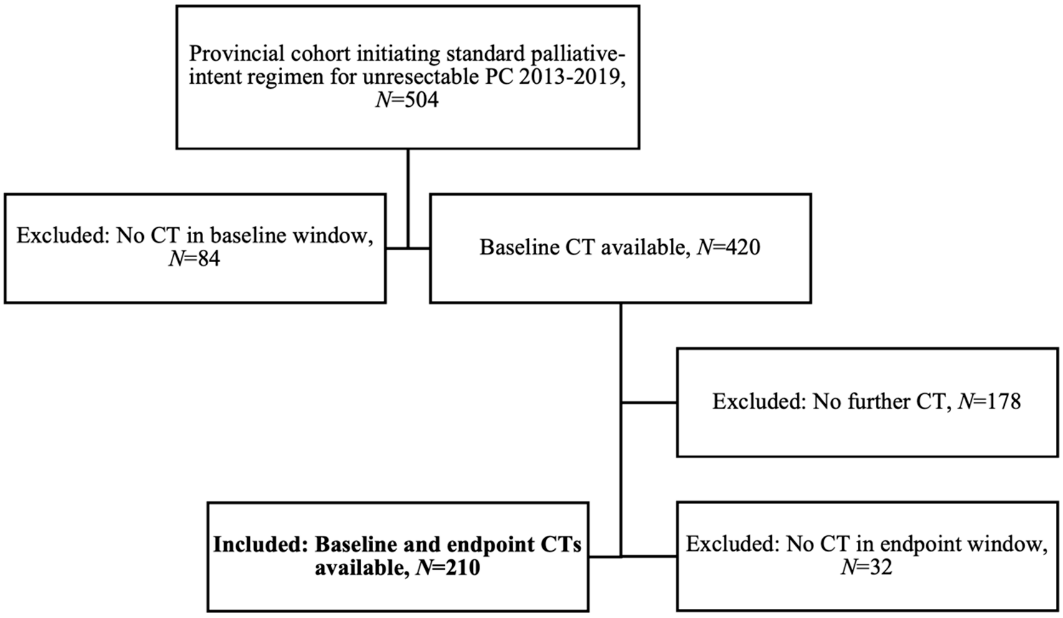


**Table S1:** Additional metrics describing skeletal muscle, adipose tissue and weight change from baseline to endpoint

| **Skeletal muscle** | **Baseline, cm²** | **Δ cm²** | **Δ Est. kg** |
|---|---|---|---|
| *Male* | 150.9 ± 27.1 | -12.1 ± 16.3 | -2.1 ± 2.8 |
| *Female* | 101.1 ± 15.0 | -5.8 ± 9.9 | -1.0 ± 1.7 |
| **Total adipose tissue** | **Baseline, cm²** | **Δ cm²** | **Δ Est. kg** |
| *Male* | 303.6 ± 155.3 | -61.5 ± 85.9 | -2.6 ± 3.6 |
| *Female* | 299.7 ± 17.7 | -59.0 ± 69.6 | -2.5 ± 2.9 |
| **Weight** | **Baseline, kg** | | **Δ kg** |
| *Male* | 80.3 ± 14.1* | | -3.1 ± 5.5 |
| *Female* | 66.0 ± 14.2 | | -2.7 ± 4.3 |

Δ $cm^2$: change in axial cross-sectional area; Δ Est. kg: Estimated total body muscle and adipose tissue changes in kilograms based on CT-defined tissue area changes at the third lumbar vertebra (L3), using regression equations published by Mourtzakis et al. 2008; skeletal muscle density 1.04 $g/cm^3$; Δ kg: weight change in kilograms based on routinely collected weight measurements taken closest to baseline and endpoint CT scans.

**Table S2:** Cox's proportional hazard model demonstrating survival impact *per -1 kg* estimated total body skeletal muscle mass and adipose tissue mass loss over a median scan interval of 115 days.

| **Characteristic** | **Univariable** | | | **Multivariable** | | |
|---|---|---|---|---|---|---|
| | **HR** | **95% CI** | ***P*** | **HR** | **95% CI** | ***P*** |
| Age *(per year)* | 0.99 | 0.98, 1.01 | .570 | n/a | n/a | n/a |
| Male sex *(vs female)* | 1.10 | 0.90, 1.59 | .212 | 0.74 | 0.54, 1.01 | .055 |
| Metastatic *(vs locally advanced)* | 1.50 | 1.11, 2.02 | .008 | 1.27 | 0.92, 1.74 | .143 |
| Tumour Progression *(vs tumour control)* | 3.04 | 2.25, 4.13 | < .001 | **2.27** | **1.64, 3.14** | **< .001** |
| Treatment after endpoint CT: | | | | | | |
| no further treatment | ref | | | ref | | |
| ongoing palliative chemotherapy | 0.29 | 0.29, 0.39 | < .001 | **0.35** | **0.25, 0.50** | **< .001** |
| curative resection | 0.06 | 0.02, 0.14 | < .001 | **0.07** | **0.02, 0.20** | **< .001** |
| Estimated total body muscle change *(per -1 kg)* | 1.15 | 1.09, 1.21 | < .001 | **1.11** | **1.04, 1.18** | **.002** |
| Estimated total body adipose change *(per -1 kg)* | 1.18 | 1.13, 1.24 | < .001 | **1.09** | **1.03, 1.15** | **.002** |

Tx: treatment; Chi-square 141.135, $P < .001$; Ref: reference; Estimated total body muscle and adipose change based on CT-defined tissue area changes at the third lumbar vertebra, using regression equations published by Mourtzakis et al. 2008; skeletal muscle density 1.04 g/cm$^3$.
